# Making teeth to order: conserved genes reveal an ancient molecular pattern in paddlefish (Actinopterygii)

**DOI:** 10.1098/rspb.2014.2700

**Published:** 2015-04-22

**Authors:** Moya M. Smith, Zerina Johanson, Thomas Butts, Rolf Ericsson, Melinda Modrell, Frank J. Tulenko, Marcus C. Davis, Gareth J. Fraser

**Affiliations:** 1 Craniofacial Development and Stem Cell Biology, King's College London Dental Institute, London, UK; 2 Department of Earth Sciences, Natural History Museum, London, UK; 3 MRC Centre for Developmental Neurobiology, King's College London, London, UK; 4 Department of Physiology, Development and Neuroscience, University of Cambridge, Cambridge, UK; 5 Department of Biology and Physics, College of Science and Mathematics, Kennesaw State University, Kennesaw, GA, USA; 6 Department of Animal and Plant Sciences, University of Sheffield, Sheffield, UK

**Keywords:** *Polyodon*, dentition, *shh*, *bmp4*, paddlefish, evolution

## Abstract

Ray-finned fishes (Actinopterygii) are the dominant vertebrate group today (+30 000 species, predominantly teleosts), with great morphological diversity, including their dentitions. How dental morphological variation evolved is best addressed by considering a range of taxa across actinopterygian phylogeny; here we examine the dentition of *Polyodon spathula* (American paddlefish), assigned to the basal group Acipenseriformes. Although teeth are present and functional in young individuals of *Polyodon*, they are completely absent in adults. Our current understanding of developmental genes operating in the dentition is primarily restricted to teleosts; we show that *shh* and *bmp4*, as highly conserved epithelial and mesenchymal genes for gnathostome tooth development, are similarly expressed at *Polyodon* tooth loci, thus extending this conserved developmental pattern within the Actinopterygii. These genes map spatio-temporal tooth initiation in *Polyodon* larvae and provide new data in both oral and pharyngeal tooth sites. Variation in cellular intensity of *shh* maps timing of tooth morphogenesis, revealing a second odontogenic wave as alternate sites within tooth rows, a dental pattern also present in more derived actinopterygians. Developmental timing for each tooth field in *Polyodon* follows a gradient, from rostral to caudal and ventral to dorsal, repeated during subsequent loss of teeth. The transitory *Polyodon* dentition is modified by cessation of tooth addition and loss. As such, *Polyodon* represents a basal actinopterygian model for the evolution of developmental novelty: initial conservation, followed by tooth loss, accommodating the adult trophic modification to filter-feeding.

## 1. Introduction

Most tooth development models reflect a bias towards morphologically derived vertebrates (e.g. zebrafish, mouse). However, more representative models for the evolution of developmental mechanisms of the dentition are provided by taxa at the base of extant vertebrate phylogenies. The basal actinopterygian order Acipenseriformes includes fossil taxa as well as the American paddlefish *Polyodon* (family Polyodontidae) and sturgeons (family Acipenseridae, e.g. *Acipenser* [1,2]) and represents an increasingly used system for addressing developmental questions in an evolutionary context [3–6]. Owing to their basal phylogenetic position, Acipenseriformes are a particularly relevant model to test hypotheses of tooth patterning and evolution. The dentition is lost in adult paddlefish and sturgeon, but present in younger individuals, although details of early stages of tooth development are poorly known [1–3,7]. As pattern order for the forming dentition has previously been described for more derived actinopterygians, comparable data for *Polyodon* will provide significant information on mechanisms in more phylogenetically basal actinopterygians.

The secreted protein sonic hedgehog (*shh*) and the TGF**-**β superfamily member bone morphogenetic protein4 (*bmp4*) are key dental patterning genes in vertebrates. *In situ* hybridization assays demonstrate that the transcripts coding for *shh*/*bmp4* are present at the earliest sites of tooth initiation with focused, time specific loci of expression restricted to dental epithelium (*shh*) [8,9] and co-expression in the underlying condensed mesenchyme (*bmp4*). Co-expression occurs on each oropharyngeal dentate field, from a diffuse band of dental competence (odontogenic band), to discrete placodes of single tooth initiation. Non**-**mammalian vertebrates for which this conserved pattern of *shh*/*bmp4* expression has been used to characterize dental patterning include a variety of teleosts (Osteichthyes, Actinopterygii): rainbow trout (*Oncorhynchus mykiss* [8,9]), Mexican tetra (*Astyanax mexicanus* [10]), zebrafish (*Danio rerio* [11,12]), several Lake Malawi cichlids [13], the freshwater pufferfish (*Monotrete abei* [14]), as well the Queensland lungfish (*Neoceratodus forsteri* [15]) and various snakes and lizards (Osteichthyes, Sarcopterygii, [16–18]) and the catshark *Scyliorhinus canicula* [19,20]. As the only non**-**teleost actinopterygian yet surveyed, our new data from *Polyodon* will provide key phylogenetic support for the hypothesis that *shh* and *bmp4* are part of a conserved and ancient gene regulatory network for patterning vertebrate dentitions.

We predict that *Polyodon* will exhibit the conserved pattern of epithelial *shh***-**positive loci, with comparable mesenchymal expression of *bmp4* [8], observed in other vertebrate taxa. Here we will use expression patterns for these genes, along with other histological and morphological datasets to demonstrate temporal differences in focal localization for each tooth site in *Polyodon,* mapping position and timing of tooth initiation to demonstrate how pattern order is established through coordinated gene activity. Our hypothesis is that this represents a basal condition of shared genetic regulation of tooth initiation times and topographic order for the Actinopterygii.

## 2. Material and methods

### (a) Animal care and sacrifice

Fertilized *Polyodon spathula* eggs were obtained from Osage Catfisheries, Inc. (Osage Beach, MO, USA) and raised to desired stages in recirculating, closed freshwater systems mimicking natural conditions (22°C, pH 7.2 ± 0.7, salinity of 1.0 ± _0.2 p.p.t. [21]). *Polyodon* were euthanized in a lethal dose of MS-222 (tricaine) and fixed for at least 24 h (dependent of tissue volume) in 4% paraformaldehyde [21].

### (b) Staging of larval *Polyodon*

*Polyodon* staging follows [3,21]: lengths for individual specimens for stages 37**–**46, and other details of the staging, can be obtained from these. Feeding larvae (beyond stage 46) are described as ‘days post-staging’ (dps) and juveniles by standard length (SL). At incubation temperature (22°C), the larval period between hatching (stage 36) and onset of exogenous feeding and yolk exhaustion (stage 46) proceeds at approximately one stage per 24 h period [21].

### (c) *In situ* hybridization

*In situ* hybridization used standard protocols [5] with riboprobes for *shh* [22] or *bmp4. Bmp4* was cloned from cDNA using the forward primer CGA GGC TAC TTT GTT GCA CA and reverse primer TCC ACG TAC AGT TCG TGT CG. Selected whole larvae (stages 41**–**45) with *shh or bmp4* expression were embedded in 20% gelatin and vibratome-sectioned at 50 μm or, embedded in 30% sucrose, frozen in liquid nitrogen and cryostat sectioned at 20 μm. Numbers of specimens (antisense, comparable number of sense), *bmp* stages 34–39 *n* = 7; 40–43 *n* = 6; 44–46 *n* = 6. *shh* stages 36 *n* = 2; 38 *n* = 3; 39 *n* = 3; 40 *n* = 2; 41 *n* = 4; 42 *n* = 2; 43 *n* = 2; 45 = 6. Photomicrographs were taken with Zeiss Nomarsky optics, or an Olympus SZX16 dissecting microscope equipped with a QImaging RetigaEXi digital camera.

### (d) Clearing and staining, CT imaging

Cleared and stained specimens (CS; Alizarin red and Alcian blue [23]) were dissected and mounted as half**-**jaws. Older specimens were studied as CS skeletal preps under a stereomicroscope and CT scanned (X**-**Tek HMX ST CT scanner, Image and Analysis Centre, Natural History Museum, London; MicroCT at Dental Institute, King's College London, GE Locus SP, creating volumes with voxel sizes 6.5 μm) and rendered using the software program Drishti (http://sf.anu.edu.au/Vizlab/drishti).

### (e) Terminology

The terms distal and proximal are used in the upper and lower jaws, with reference to the jaw joint (proximal) and symphysis (distal). The terms rostral and caudal, dorsal and ventral are used with respect to the body axes.

## 3. Results

**Figure 1.**
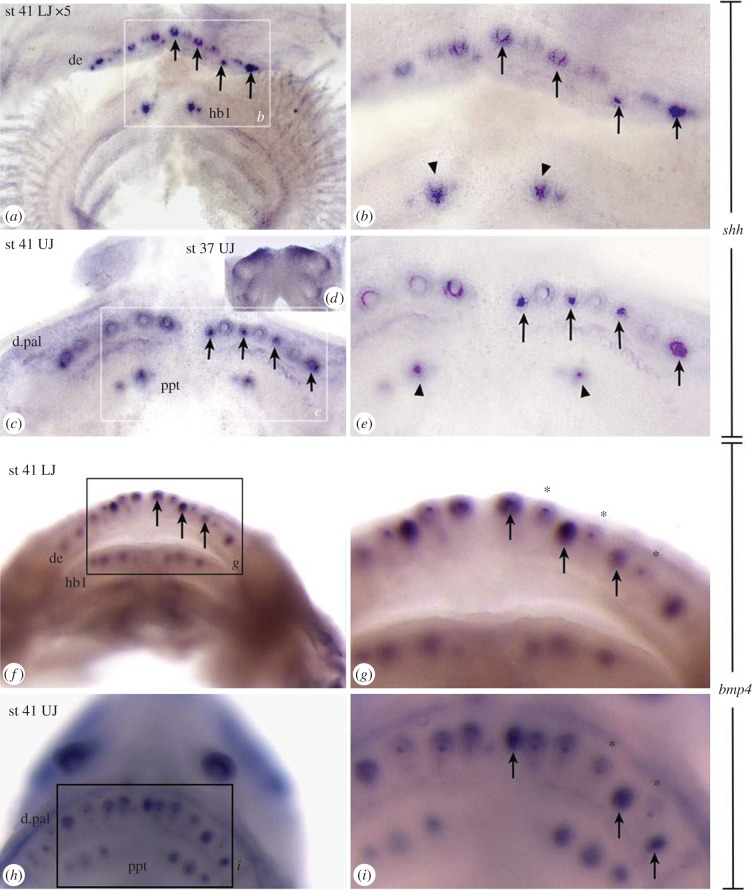
Expression of *shh*, *bmp4* in *Polyodon spathula* oral and pharyngeal initial dentitions, stage 41. (*a–c,e*) *shh* expression in tooth buds of cleared whole mount jaws compared with (*d*) stage 37 upper jaw, expression restricted to oral surfaces and on first infrapharygobranchial arches. (*a,c*) Multiple loci on tooth fields of dentary and dermopalatine, only two loci on hypobranchial and palatopterygoid. Arrows indicate alternate timing of strongest expression. (*b,e*) Strong expression in hypobranchial 1 and palatopterygoid (arrowheads); cone expression in dentary, hypobranchial, dermopalatine, compared to early placode expression on palatopterygoid. (*f–i*) *bmp4* expression for comparison to *shh* expression. (*f,g*) Lower jaw, (*h,i*) upper jaw *bmp4* in the dental papillary mesenchyme marks all oral jaw tooth positions. Dental mesenchyme underlies the dental epithelium and expression appears diffuse, however, more intense expression is seen at alternate tooth loci (arrows, *f*,*g,i*) with weaker expression indicating earlier (older) loci (asterisk), equivalent to *shh* expression pattern. Abbreviations: b1, 2, basibranchials; ba, bone of attachment, cb1–5, ceratobranchials; ch, ceratohyal; de, dentary; d.pal, dermopalatine; hb1, 2, 1st, 2nd hypobranchial; hb1tp, hb2tp, hypobranchial toothplates; hh, hypohyal; hym, hyomandibular; itg, incipient tooth germ; iph, infrapharyngobranchial; iphtp, infrapharyngobranchial toothplate; Mc, Meckels's cartilage; ppt, palatopterygoid; tc, tooth cone; 2ndt, second tooth.

**Figure 2.**
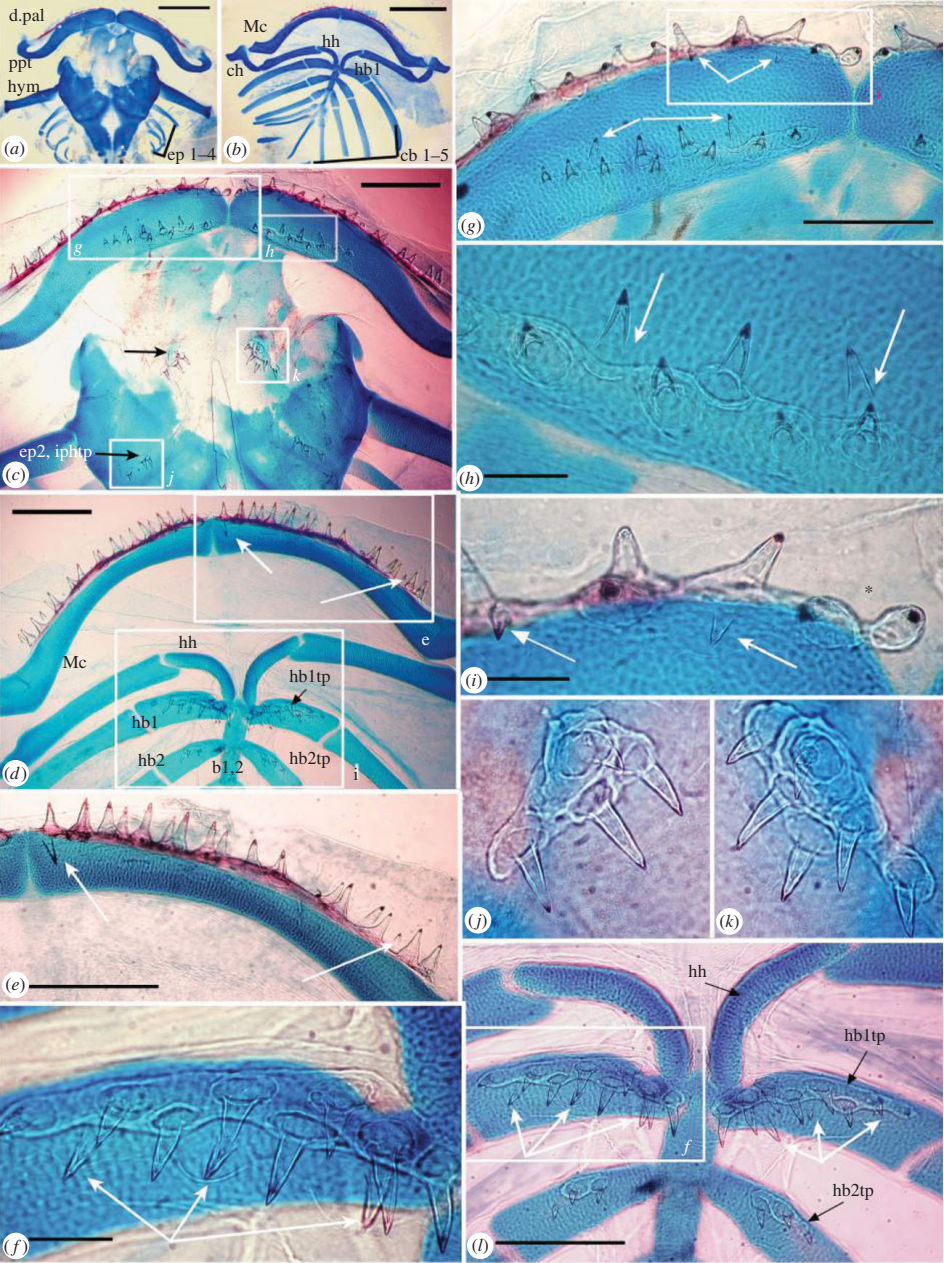
Alizarin red, Alcian blue preparations of *Polyodon spathula*, 7dps showing relative tooth positions. (*a, c, g-k*) Upper jaw and dorsal pharyngeal skeleton, (*b*,*d–f,l*) lower jaw and ventral pharyngeal skeleton. (*a,b*) Chondrocranium and branchial arches. (*c*) Upper jaw, teeth along dermopalatine bone and separate palatopterygoid tooth plate (lacking membrane bone), with two paired tooth plates caudally (black arrows indicate *j, k*). (*d*) Lower jaw, ventral pharyngeal skeleton (hyoid, 1st, 2nd gill arches). (*e*) Teeth on dentary bone (arrows, new teeth). (*f*) Eight teeth linked by bone of attachment on 1st gill arch cartilage (hypobranchial 1, lacking membrane bone). (*g–i*) Right upper jaw, teeth ankylosed to dermopalatine bone, separate palatopterygoid tooth plate (arrows, new teeth caudally on dermopalatine (*i*), rostrally on palatopterygoid (*h*)). (*h*) Palatopterygoid tooth plate, bone of attachment only (arrows new teeth). (*j,k*) Upper jaw tooth plates of (*j*) epibranchial 2, four associated teeth, (*k*) hyoid arch, six teeth. (*l*) Hypohyal and first two ventral gill arches, with paired toothplates, more teeth on hb1 than hb2, more on ventral than dorsal pharyngeal toothplates. White arrows = newest unattached teeth. Scale bars (*a,b*), 1 mm; (*c–g,l*), 500 μm; (*h,i*), 100 μm; abbreviations as in figure 1.

In *P. spathula* larvae, *shh* and *bmp4* expression reveal both the early events of oral and pharyngeal dental patterning and sequential addition of tooth loci as development proceeds. There are notable differences in the addition of new tooth germs in individual dentate fields, normally caudal, but exceptionally rostrally on the palatopterygoid tooth plate. Concerning timing along the body axis, tooth initiation begins in association with Meckel's cartilage, establishing a spatio**-**temporal gradient that extends from the oral, through to tooth sites in the pharyngeal cavities (figures 1 and 2; electronic supplementary material, figure S4). Skeletal preparations provide additional data on pattern order; after tooth rows form on the dentary and dermopalatine, they develop on the more caudal palatopterygoids and first hypobranchials (figures 1*a*,*c* and 2*a,b*, respectively). Teeth are later organized into toothed plates, connected by basal bone of attachment, representing functional surfaces of the oropharyngeal dentition (table 1, electronic supplementary material, figure S2*c*) [1–3].

**Table 1.** Rostro-caudal and ventro-dorsal graded trends from oral to pharyngeal sites in tooth addition during development and transition of the embryo to juvenile dentition, *Polyodon spathula. Differences in total tooth number at each stage of development are shown and reflect a directed pattern in time and space throughout the oropharyngeal cavity.* Abbreviations: de, dentary; d.pal, dermopalatine; epb, epibranchial; hb1, 2, hypobranchial 1, 2; iph, infrapharyngobranchial; ppt, pterygopalatine; UBS, LBS, upper, lower branchial skeleton; UJ, LJ, upper, lower jaw. Numbers are per left or right half.

specimen	UJ d.pal	UJ ppt	LJ de	UBS iph	UBS epb	LBS hb1	LBS hb2	figure number
St. 39–40 *shh*	4 + 1	0	5	0	0	0	0	electronic supplementary material, S4*c–h*
St. 41–42 *shh*	4 + 2	1 + 1	7	0	0	1 + 1	0	1; electronic supplementary material, S4*a, i–n*
St. 43 shh	8 +	2	11+	0	0	1	0	4*o,p*
stage 45 *shh/bmp*	14	8	16–18	2	0	4	0–1	3; electronic supplementary material, S6
*7dps larva*^a^	*17–20*	*9–11*	*21–22*	*4–6*	*4*	*11*	*3–4*	2
*TL–345 mm*^b^	*55*	*55*	*91*	*0*	*0*	*30*	*10*	electronic supplementary material, S1 and 2

^a^Based on *n* = 5 cleared and stained whole mount.

^b^Based on CT scan data.

### (a) Timing of *shh* expression in whole mounts maps sequential tooth initiation (stages 37–43)

**Figure 3.**
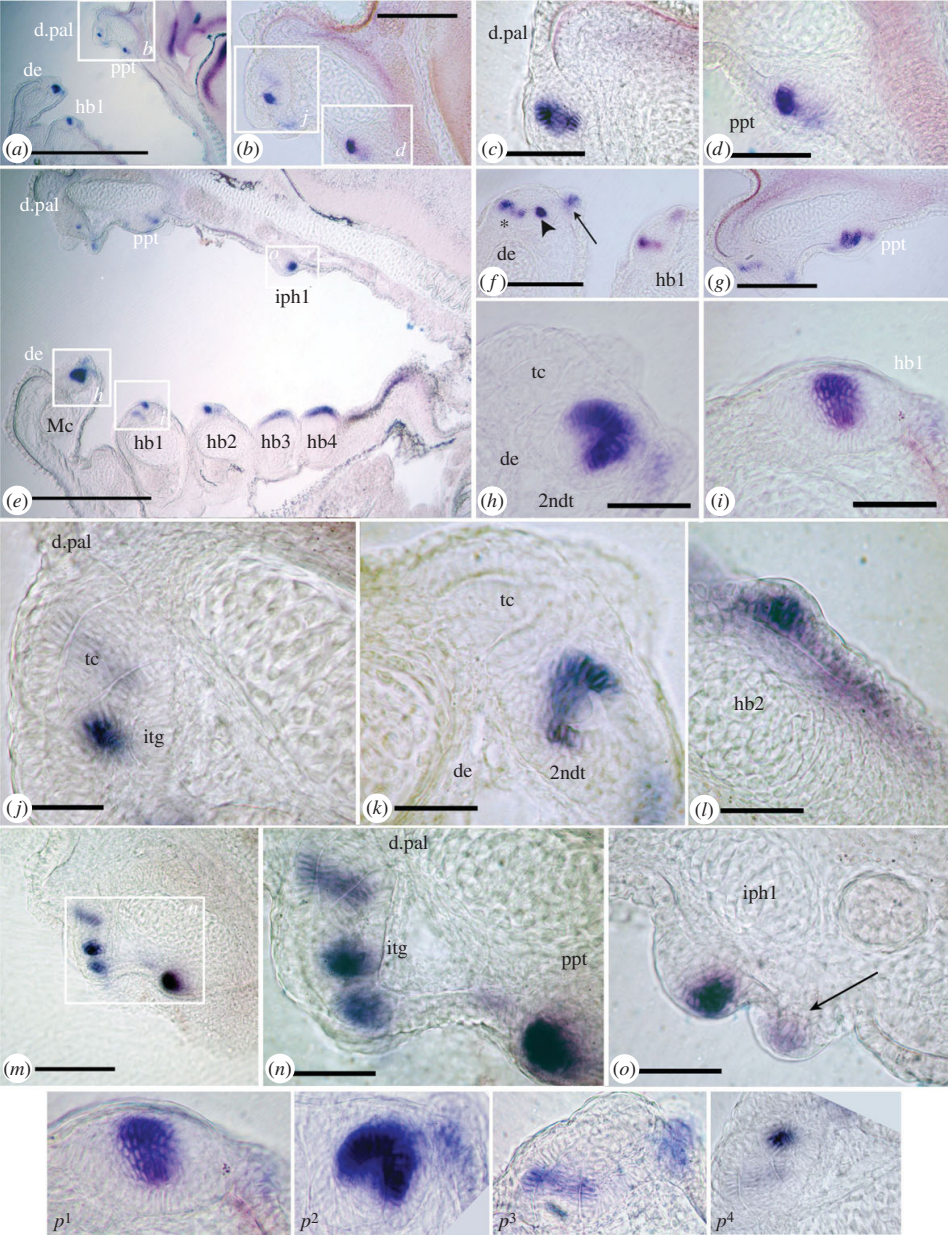
Serial sagittal sections, *Polyodon spathula* (stage 45) after *in situ* hybridization for *shh* show sequence of tooth morphogenesis*.* Photomicrographs, low and high magnification (objectives 6.3×, 16×, 40×) of location and rostro-caudal timing of *shh* gene expression in all tooth fields relative to tooth germ morphogenesis, rostral, left and dorsal, top. (*a–d*) Most medial section, expression in dermopalatine (cone + collar, *p*^3^) and palatopterygoid (placode, *p*^1^). (*e*) More lateral section including Meckel's cartilage and pharyngeal arches. Expression loci associated with first stages of morphogenesis (placode, *p*^1^) on the 1st upper branchial arch (iph1), 1st and 2nd hypobranchials. By comparison, on 3rd and 4th pharyngeal arches tooth bud foci absent, localization is a field of expression, a stage prior to tooth morphogenesis. (*f*) Low magnification field of variation in expression loci on dentary and hypobranchial1, with collar epithelium downregulated on first tooth (asterisk) and adjacent second tooth germ shown as intense expression (arrowhead, weak expression in sensory papilla, arrow as (*o*, *p4*). (*g*) Low magnification view of variation in expression at loci on the dermopalatine (downregulated) and palatopterygoid strong expression in all dental epithelium around dentine cone (late cap stage). (*h*) Tooth cone (tc) developed, and 2nd tooth germ (2ndt) at cap stage (*p*^2^). (*i*) First hypobranchial, placode stage of *shh* expression (*p*^1^). (*j*) Tooth cone with second incipient tooth germ (itg), strong expression (*p*^4^). (*k*) Downregulation from cap to ‘collar’ expression (*p*^3^) in 2nd tooth. (*l*) Early tooth placode in oral epithelium of 2nd hypobranchial. (*m*) Upper jaw palatoquadrate cartilage with tooth germs on dermopalatine and palatopterygoid at different morphogenetic stages. (*n*) Four stages of *shh* expression, tooth cone with downregulated expression, incipient second tooth germ on dermoplatine, on palatopterygoid, cap stage. (*o*) Infrapharyngobranchial (iph1) upregulated strong expression (note evaginated tooth germ, placode-cap), alongside weak expression in sensory papilla (arrow). (*p*^1*–*4^) Four stages of *shh* expression in tooth germs, oral epithelium dorsal, contrast enhanced (translated into diagram as figure 4*a–d*). Scale bars (*a,e*), 250 μm; (*b,f,g,m*), 50 μm; (*c*,*d*,*h–l*,*n–p*^1*–*4^), 25 μm; abbreviations as in figure 1.

**Figure 4.**
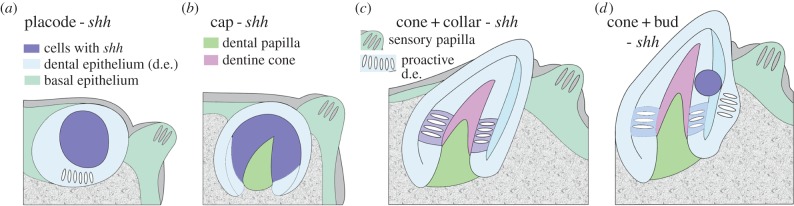
Diagram summarizing stages of tooth germ morphogenesis relative to *shh* expression (from figure 3*p*^1–4^). Intensity of cellular expression is partitioned characteristically within the dental epithelium, with negative differentiated, interactive cells of dental epithelium shown (proactive d.e.), and also in sensory papilla of taste buds on right of tooth germ. (*a*) Cellular partitioning of *shh* expression as ‘placode’ (localized within epithelium, can be evaginated). (*b*) ‘Cap’, expression in the cap-shaped epithelium of tooth germ, surrounding dental papilla. (*c*) ‘Cone + collar’, cones of dentine with expression associated with the tooth base, or collar epithelium below the cap. (*d*) ‘Cone + bud’, expression in a new site within the outer dental epithelia (incipient bud for new tooth germ).

Spatial expression of *shh* occurs as focal loci, with changes in intensity coincident with each stage of tooth germ morphogenesis, mapping location and developmental timing for each tooth position (figures 1, 3 and 4). This pattern of spatio**-**temporal expression identifies new tooth germs added relative to preexisting ones, in precise locations at sequential times, from one dentate region to another (table 1).

*Shh* expression is first observed in the odontogenic fields beginning at stage 37 (figure 1*d*; electronic supplementary material, figure S4*b*). Strong expression loci on the odontogenic band occur first as focused placodes (stages 39**–**41; figure 1*a–e*), then expression as a cap around the cone of the tooth tip (figure 3*p*^2^ and 4*b*; electronic supplementary material, figure S4*c–h*). These loci mark tooth positions within one row (figure 1; electronic supplementary material, figure S4*i–p*). *Shh* expression is next upregulated at alternate (second) tooth positions, within this same row (figure 1*a–c,e*, arrows). By stage 43, *shh* is downregulated in epithelial cells of older tooth germs around tooth cones. Accurate counts of tooth number from *shh* expression at these later stages relies on seeing tooth cones (using Nomarsky optics). Nevertheless, differences in total number between upper and lower jaws are observed (table 1); for example, at stages 40 and 42 there are more tooth loci on the dentary than dermopalatine (compare electronic supplementary material, figure S4*g,h* (new parasymphysial tooth on dentary) with figure 4*e,f* and *i–m* (new loci added distally) with *n*).

Given this recognizable developmental sequence of epithelial *shh* expression, sites of tooth initiation can be identified along the rostro-caudal body axis. In both jaws at stages 39–40, there are four to five tooth buds in each dentary and dermopalatine field, contrasting with lack of tooth buds in more caudal toothed sites (electronic supplementary material, figure S4*c*–*h*). Later, at stage 41 the dentary and dermopalatine have seven tooth positions with alternating higher intensity of *shh* expression, and a new distal and proximal tooth germ, all in the same tooth row (figure 1*e,i*, arrows). As well, two *shh***-**positive tooth loci are present on the first hypobranchial and the palatopterygoids (figure 1*a–c,e*, arrowheads). In stages 42–43, these *shh* expression sites are intense caps around the tooth cone (electronic supplementary material, figures S4l*a* and l*b*), forming rings in later stages where *shh* is downregulated in cap cells (electronic supplementary material, figure S4*o–p*, further details see §3*c,d* and figure 4).

### (b) *bmp4 e*xpression maps timing of cooperative activity during tooth morphogenesis (stages 40–45, 1dps)

All stages show *bmp4* expression associated with each tooth locus (figure 1*f–i*; electronic supplementary material, figure S5). When compared to stage-matched specimens stained for *shh*, intense expression of *bmp4* appears associated with mesenchyme of the newest forming tooth loci (figure 1*g,i*, arrows). Notably, stage 41 and 45 *bmp4* expression shows upregulation in alternate positions of (second) tooth germs within the tooth row, on the dentary and dermopalatine, while the most rostral (first) tooth germs are dentine cones with *bmp4* downregulated in the papilla. Note these show strong papillary expression in more caudal sites, indicating that these are younger (figure 1*f,g,i*; asterisk versus arrows, respectively). However, on the palatopterygoid, the intense papillary *bmp4* expression of the younger loci is rostral to the dentine cones, as observed in the expression pattern for *shh* (i.e. an opposite second tooth addition pattern to the dentary and dermopalatine, electronic supplementary material, figure S5*h’*, st 42, 5*l’*, st 45, arrows.

### (c) Cellular expression of *shh* during tooth germ morphogenesis, stage 45

The exact location of expression within the epithelial tooth germ is shown in more detail in serial, parasagittal sections than in whole mount *in situ* (figure 3; electronic supplementary material, figure S4), while the mesenchyme of the dental papilla shows complimentary *bmp4 expression* (electronic supplementary material, figure S6). Gene expression changes are associated with different tooth germ morphologies through development (figures 3*p* and 4), where different intensities are associated with specific timing of morphogenesis at each tooth site in the oropharyngeal cavity, including first locations of the sites on the branchial arches. These demonstrate a rostro**-**caudal activation gradient of tooth initiation for each dentate field. Initially, the placode shows intense *shh* and *bmp4* expression and is superficial (no dental lamina), with *shh* located to the middle epithelial cells (figure 3*d*,*i*,*p*^1^). In the cap stage, *shh* is more intense in all epithelia, surrounding the papilla (figure 3*g*,*p*^2^; *bmp4*, electronic supplementary material, figure S6*b*). After dentine histogenesis, *shh* is downregulated in the cap cells but is strongly expressed in the epithelium as a collar around the tooth cone (cone + collar stage, figures 3*c,p*^3^, 4*c*). Subsequently, *shh* is downregulated around the whole tooth cone (figure 3*j,n,p*^4^), but within the adjacent dental epithelium (not the inner dental epithelium), *shh* is upregulated as an intense focal expression, attributed to an incipient, successive tooth germ (figures 3*j,n*, 4*d*; electronic supplementary material, figure S6*a*,*c,d*). In the second, alternate tooth position the same steps of *shh* expression are observed, including cap and cone + collar stages (figure 3*f,h,k*).

Serial sections show these expression stages simultaneously throughout the oropharyngeal cavity. Loci of *shh* expression occur dorsally on the dermopalatine and palatopterygoid (figure 3*a–d,g*), and ventrally on the dentary and 1st hypobranchial (figure 3*e,h,i*; electronic supplementary material, figure S6*a*), along with a focal spot on the infrapharyngobranchials dorsally and 1st and 2nd hypobranchials ventrally (figure 3*e,h,i*; electronic supplementary material, figure S6*a,d*), but a field of expression on the more caudal branchial arches (figure 3*e*). When dentine is present in the first dentary teeth, as a collar plus translucent cone, the more caudal, second tooth germ is only at the placode stage (figure 3*f*). In other sections, the first tooth appears as a translucent dentine cone with a second tooth at cap, or collar stage (figure 3*h*). All these observations show a staggered time difference in each second tooth germ, as well as the first (*bmp4* data, electronic supplementary material, figure S6*c,d*). Similar staggered stages are seen in the dermopalatine tooth germs, and those of the palatopterygoid relative to the dermopalatine (figure 3*m,n,g*).

The restriction of *shh* expression to an intense focal locus (placode) forms first in the evaginated epithelium above the cartilage on the 2nd, as in the 1st, hypobranchial (figure 3*l*). The placode is superficial (i.e. forms without a dental lamina; figure 3*n,o*,*p*^1^), but also evaginated at the cone-cap stages (figure 3*h*,*p*^2^), then just within the expanded dental epithelium at cone + collar stage (figure 3*k,n*,*p*^3^). When *shh* is downregulated in all dental epithelium around the tooth there is an upregulated intense locus of *shh* expression next to this first tooth, in the dental epithelium, ‘cone + bud’, not evaginated but located in the epithelium adjacent to the dentine cone. Papillae with taste buds on the inner oral epithelium always exhibit faint *shh* expression, similar in intensity to the downregulated collar epithelium (figures 3*o*, arrow and 4*c*, sensory papilla with differentiated cells), while *bmp4* expression is absent (electronic supplementary material, figure S6).

### (d) Skeletal preparations show tooth addition positions in 7dps larvae

#### (i) Tooth development on upper jaw, dorsal branchial skeleton

Tooth rows are present ventral to the upper jaw cartilage, both rostrally on the dermopalatine bone and caudally on the palatopterygoid. The dermopalatine has 17–20 ankylosed teeth, while the latter lacks an independent ossification at this stage, with teeth conjoined by the individual bone of attachment of each tooth (translucent rings, figure 2*a,c,g,h*). Caudal to the palatopterygoid are two paired patches of teeth, the first associated with the hyoid arch with six teeth, joined only by their bases (figure 2*c*, black arrow, white box, *k*). The second is associated with the second infrapharyngobranchial, possessing four teeth (figure 2*c*, white box, *j*). The dermopalatine bone represents the most developmentally advanced in the upper jaw with new unattached teeth being added caudal to tooth positions 2 and 4, as well as parasymphysially (arrows, figure 2*g,i*). On the ventral surface of the palatopterygoid cartilage, the oldest teeth are joined together via attachment bone (dentine cones expanded into cylinders), with 11 teeth on the right side, nine on the left. As opposed to the caudal tooth addition associated with the dermopalatine, two new teeth (lacking bony rings; figure 1*g,h*, arrows) are rostral to the attached (older) teeth.

#### (ii) Tooth development on lower jaw, ventral branchial skeleton

Tooth rows are present dorsally on Meckel's cartilage, with 22 left and 21 right teeth fused to the dentary bone via bone of attachment with new, unattached teeth caudal to the attached (older) teeth and at proximal and distal ends of the row (Mc, figure 2*d,e*, arrows). Other toothed plates are caudal to Meckel's cartilage in the pharyngeal cavity, on the hypobranchials (first, 11 teeth; second, three teeth). Hypobranchial teeth are not ankylosed to bone but older teeth are joined at their bases via their individual bone of attachment (figure 2*d,f,l*). Three new teeth (not joined by bone of attachment) on left hypobranchial 1 are added caudally (figure 1*f*, arrows). By later functional stages, with increasing tooth numbers at all sites, pharyngeal teeth are arranged in radial rows (four to five teeth in each), differing from the oral dentition (electronic supplementary material, figure S2*a,b*).

## 4. Discussion

Combined data from ontogenetic stages of *P. spathula* establishes sequences of gene expression and tooth morphogenesis in the oropharyngeal cavity, allowing spatio-temporal patterns of tooth initiation and development to be documented; tooth rows form on the dentary and dermoplatine before the more caudal palatopterygoids and first hypobranchials (figures 1*a*,*c* and 2*a,b*, respectively). Teeth are later organized into toothed plates, connected together by basal bone of attachment, independently of the membrane bone, representing early functional surfaces of the oropharyngeal dentition (electronic supplementary material, figure S2*c*). Skeletal whole mounts show where new teeth are added to individual dentate fields, while post**-**larval stages indicate that tooth addition slows and teeth are lost (electronic supplementary material).

These observations indicate progressive rostral–caudal and ventro-dorsal tooth initiation/addition gradients within the oropharyngeal cavity: tooth addition occurs first on Meckel's cartilage, showing alternate patterns of gene expression along the tooth row, prior to the dermopalatine (stage 40, electronic supplementary material, figure S4*g*,*h*; stage 42, electronic supplementary material, figure S4*i*–*m* versus *n*). At 7dps, a larger number of teeth are present on the dentary (figure 2) and at later juvenile stages the dentary shows substantial toothless areas of membrane bone relative to other dentate regions in the oropharyngeal cavity, due to tooth-related loss of attachment bone (electronic supplementary material, figures S1*f–i* and S3*c–f*, asterisk). With respect to a rostral–caudal gradient of tooth addition, the dentary and dermopalatine develop tooth germs with a cone of dentine before the palatopterygoid (electronic supplementary material, figure S5), while teeth in the oral cavity develop before those in the pharyngeal cavity. There is also a rostral–caudal progression in the pharyngeal cavity with the placode stage attained in hypobranchial 1, versus field expression on hypobranchials 2. The former has the most teeth; caudally hypobranchials 3 and 4 never show upregulated tooth loci. With respect to rostro-caudal tooth addition on each oral site, new tooth buds are initiated caudally on the dermopalatine and the dentary (figure 1*i*, electronic supplementary material, figure S5*m,n*), but new teeth form rostrally on the palatopterygoid (figure 2g*,h*).

Our results show that *shh* and *bmp4* expression data during *Polyodon* tooth initiation follows the same spatio-temporal order observed in all other non-mammalian vertebrate species assayed to date [8,9,14–17,24]; however, our observations on the ordered sequence of timing of tooth germ initiation in oral and pharyngeal tooth sets also reveal directed rostro-caudal and ventro-dorsal patterns. This graded progression has not previously been reported for actinopterygians, or for gnathostome oropharyngeal dentitions. Nevertheless, tooth patterning, at least with respect to tooth initiation and differentiation appears evolutionarily stable and highly conserved among gnathostomes. For example, no differences in collocation of *shh* and *bmp4* expression were detected between developing oral and pharyngeal teeth in *Polyodon*, comparable to a variety of other taxa. Along with the ordered tooth initiation sequence, this implies that tooth germs in all regions are equivalent and conserved modular vertebrate units.

We have demonstrated cellular partitioning for *shh* and *bmp4* expression and sequential stages of tooth germ morphogenesis from ‘placode’, ‘cap’, ‘cone + collar’ to ‘cone + bud’ (figure 4). This is based on expression intensity that changes in a characteristic sequence within the dental epithelium, for each developing tooth germ. Notably, a new locus for strong expression forms alongside the developed, functional tooth (‘cone + bud’). We interpret this as the incipient tooth germ representing what we term a successional tooth. This is distinct from superficial, initial tooth ‘placodes' and is consistent with observations that in actinopterygian fish, successional teeth form from the older tooth and not from a dental lamina [8]. In some actinopterygian taxa (Cyprinidae, derived teleosts), functional and replacement teeth can be retained as a pair, particularly during larval stages, although the functional tooth is eventually lost with the replacement tooth moving into place [25]. In *Polyodon*, by comparison, the functional tooth is retained and not lost in response to the presence of the successional tooth; the latter should therefore not be considered a replacement tooth *per se*. Tooth loss occurs much later in *Polyodon* in what appears to be a general reduction and loss in the oropharyngeal cavity. This suggests that the more typical osteichthyan dentition pattern, with tooth replacement, never happens and is altered at this early ontogenetic stage.

Despite the enormous diversity, the presence of teeth organized into a functional dentition is a shared feature among jawed vertebrates, undoubtedly one reason for their evolutionary success, allowing a variety of feeding niches to be exploited. This diversity is underpinned by a high degree of developmental genetic conservation, particularly in early development, in taxa such as trout [7,8], cichlids [10,24] and the pufferfish [12]; these early patterns are also seen in sarcopterygian fish *Neoceratodus* [13] as well as the shark *Scyliorhinus* [17,26]. This conservation is also present in the dentition of *P. spathula*, with modifications early in development, including tooth retention and lack of replacement teeth. Tooth addition slows, while in *Acipenser*, teeth are lost, entirely linked to suction feeding adaptations [1,2]. However, we currently lack information on candidate genes involved in tooth regeneration that may change, or be missing in *Polyodon* and *Acipenser* [7]; other basal taxa, such as *Polypterus*, show full dentitions with tooth replacement [27]. New analysis of genes directed towards key transitions from tooth initiation to replacement in *P. spathula* will offer insight into the evolution of tooth regeneration strategies and dental diversity. Modifications to the dentition that occur later in ontogeny, allow the diversity of vertebrate dentitions to be expressed [10], and are the precursor steps to the development of drastically different modes of feeding among the gnathostomes.

## Supplementary Material

Making teeth to order: conserved genes reveal an ancient molecular pattern in paddlefish (Actinopterygii)

## Data accessibility

Drishti files for scans of *Polyodon spathula* are available at http://chondrichthyes.myspecies.info/.
