# Supplementary material for: Making teeth to order: conserved genes reveal an ancient molecular pattern in paddlefish (Actinopterygii)
Source: Proc Biol Sci. 2015 Apr 22;282(1805):20142700. doi: 10.1098/rspb.2014.2700 (PMC4389609; doi:10.1098/rspb.2014.2700)
Supplement: Making teeth to order: conserved genes reveal an ancient molecular pattern in paddlefish (Actinopterygii) [file rspb20142700supp1.pdf]

Electronic Supplementary Material

Making teeth to order: conserved genes reveal an ancient molecular pattern in paddlefish (Actinopterygii)

Moya M. Smith[a,b], Zerina Johanson[b], Thomas Butts[c], Rolf Ericsson[b], Melinda Modrell[d], Frank J. Tulenko[e], Marcus C. Davis[e], and Gareth J. Fraser[f]

Development of the acipenseriform dentition was previously described for *Polyodon* and *Acipenser* [1-3, 7]. In *Polyodon*, the teeth and jaws were said to be among the first elements to ossify [1, 29mm TL specimen; 7, 18.8mm TL specimen of *Acipenser*], but the relative size of the teeth decreased as body size increased, being absent in larger individuals. It was suggested that no further teeth were added, and that some teeth were shed, while others were overgrown by the bones of the jaw [1, 2]. Teeth are lost even earlier in ontogeny in *Acipenser*, from the upper jaw by 42mm TL and from the lower by 27.6mm TL; there was no indication that teeth in *Acipenser* were incorporated into the growing jaw [7]. Teeth are also present on the gill arches and are the last to be lost in *Acipenser* (by 50mm TL [7]), but are described as “lost or highly reduced in relative size in large specimens” in *Polyodon* [1]. Here, we focus on juvenile, sub-adult specimens that still show relatively complete dentitions although tooth addition appears to have ceased and with tooth and bone loss occurring (electronic supplementary material ESM figures 1-3).

Results

In the oral dentition of *Polyodon* teeth are present on the dermopalatine and palatopterygoid of the upper jaw and the dentary of the lower jaw (electronic supplementary material, figures 1, 3). As well, there are pharyngeal toothplates on the anterior elements of the gill arches, first and second only (electronic supplementary material, figure 2, hb1, hb2). Although *shh* was expressed as odontogenic field in early ontogenetic stages, teeth have never formed (figure 3). In both early and later ontogenetic stages, there are fewer teeth associated with the oral and pharyngeal dentitions, compared to other basal actinopterygians such as *Polypterus* [27] and *Amia* [28].

Skeletal preparations show cessation of tooth addition in juvenile *Polyodon*

In cleared and stained specimens of a juvenile *Polyodon* (34.5cm TL), teeth are present on both the upper and lower jaws in greater numbers compared to earlier ontogenetic stages, new teeth having been added distally, after the first two alternate rows that characterize the embryonic dentition. Tooth addition still occurs, for example, rostrally on the palatopterygoid (electronic supplementary material, figure 1*e*, white arrow). However, in older specimens, any pattern to the addition of new oral teeth appears to have been lost. For example, scattered teeth are present (electronic supplementary material, figure 1*a, c*) and lack the organization seen in 1-day post staging individuals, where they are time-staggered alternately (figure 1*e, f, g-i*).

New teeth are rare in the oral cavity in juvenile stages, but more clearly present in association with the pharyngeal dentition, and more clearly patterned, relative to the oral dentition (electronic supplementary material, figure 2*d*, black arrows, arrowhead). Hypobranchial tooth plates have more teeth than on the 7dps specimen but lack any membrane bone (electronic supplementary material, figures 2*c*, 3*c*).

The oral jaws are characterized by extensive toothless rings of bone, and additional bone loss around the tooth; (electronic supplementary material, figure 1*c, g, h*, arrowheads). In electronic supplementary material, figure 1*h*, there are three regions of bone loss, increasing in size and becoming more elongate, from distal to proximal. In some regions tooth absence is nearly complete, particularly associated with the dermopalatine and dentary (electronic supplementary material, figures 2*d*, associated with isolated tooth tips, arrowhead, 3*c*, asterisk).

Discussion

Tooth patterning and addition is maintained in the juvenile pharyngeal toothplates , with teeth on the hypobranchial toothplates organized in radial rows, and new teeth added both to the caudal and proximal sides of each row (figure 1*f*; electronic supplementary material figure 2). The arrowhead in electronic supplementary material figure 2*d* indicates a new distally added tooth as part of the outer row, with two new being added caudally (black arrows).

In contrast, the upper and lower oral jaws are characterized by tooth loss, represented by the open bony rings and areas of bone loss. Tooth addition is characterised by tooth initiation and development, with subsequent bone of attachment development, and finally mineralization of this bone and the associated toothplate (figure 2). Given this sequence, we suggest that the open rings seen in the juvenile oral dentition represent loss (rather than tooth addition), first of the tooth itself via resorption of the bone of attachment, forming the rings within the toothplate; additional bone resorption creates the large open spaces along the jaws (electronic supplementary material figure 1*g, h,* arrowheads). In the palatopterygoid of the upper jaw, two teeth have the appearance of being

resorbed and lost from the toothplate as an initial stage of this process (electronic supplementary material figure 1*e*, arrowhead, small black arrow).

More rostral teeth appear deeper to the surface, sunken in pits, where either bone loss has occurred around the tooth as part of the initial stages of tooth loss described above (electronic supplementary material, figure 1*i*, arrowheads); alternatively this could represent initial stages of the incorporation into the dermal jaw bone, as previously described [1]. Examination of CT-scan individual virtual slices however, shows no indication that this incorporation via bone overgrowth has occurred.

Although *Polyodon* displays initial conservation of tooth addition to the jaws, pattern order is soon lost, followed by gradual loss of teeth, presumed to be via resorption of bone of attachment. Loss of teeth from the oral cavity prior to the pharyngeal cavity replicates the rostrocaudal sequence of tooth addition described in the main text. Our ongoing project will focus on the role of osteoclasts in resorbing the *Polyodon* dentition (e.g., via TRAP). There is no indication of successive, ongoing tooth replacement, compared to continual tooth replacement in taxa such as the catshark and teleosts (trout and cichlids) and the squamates [6, 7, 9, 12, 16]. Tooth replacement occurs even in derived teleosts such as the pufferfish, although highly modified and controlled by the symphyseal odontogenic tissues [10]. Future work will investigate how the tooth replacement mechanism is modified/lost in *Polyodon* such that information on the genetic modulation for this loss may suggest a mechanism for how reduction in tooth replacement occurs throughout the vertebrate phylogeny, including mammals, with only one set of successor teeth.

Additional references

28 Grande, L. and Bemis, W. E. 1998 A comprehensive phylogenetic study of amiid fishes (Amiidae) based on comparative skeletal anatomy: An empirical search for interconnected patterns of natural history. *Soc. Vert. Paleo. Mem*. **4(i–x)**, 1–690.

Electronic Supplementary Material

Figure 1. *Polyodon spathula*, American Museum of Natural History AMNH 39150 (36.9cm SL). *(a)* upper and lower jaws in articulation, upper jaw dentition visible. *(b)* lower jaw removed, upper dentition including dermopalatine and palatopterygoid. *(c)* closeup of dermopalatine and palatopterygoid dentitions, arrowhead indicates area of potential bone and tooth loss. *(d)* closeup of dermopalatine dentition showing regions of bone and tooth loss, arrowhead indicates remaining tooth tips. *(e)* closeup of palatopterygoid dentition, small black arrow and arrowhead indicate teeth in the process of being lost, white arrow shows new tooth being added. *(f)* Meckel's cartilage and dentary bone in dorsal view showing teeth along margin. *(g, h)* closeup of dentary bones near the symphysis, arrowheads indicate areas of more extensive bone and tooth loss, (i) closeup of dentary, arrowheads show areas of potential bone loss around individual teeth. Abbreviations as in figure 1.

Figure 2. *Polyodon spathula*, American Museum of Natural History (AMNH) 39150 (36.9cm SL). *(a)* lower gill arches in dorsal view showing gill rakers and toothplates rostrally. *(b)* closeup of hypobranchial toothplates, in hypobranchal 1, there appear to be 4 tooth rows near the jaw symphysis, with a reduced number of rows laterally along the gill arch cartilage. *(c)* closeup of hypobranchial toothplate 2 (the toothplate

on the opposing hypobranchial is absent and presumed lost. *(d)* closeup of hypobranchial toothplate 1, lateral teeth showing alternate tooth addition, arrows and arrowhead indicates new teeth being added. Abbreviations as in figure 1.

Figure 3. *Polyodon spathula (a)* Field Museum of Natural History FMNH99272 (Grande and Bemis 1991: fig. 12B), specimen in lateral view. *(b, c)* Natural History Museum, London Zoology collection 89.6.8.82 (32.3cm SL), CT-scan of skull and gill arches *(b)* lateral view of skull, *(c)* ventral view of articulated upper and lower jaws, with gill arch toothplates. *(d-f)* American Museum of Natural History AMNH88849 (79cm, length estimated), skeletal preparation, lower jaw, CT-scan, scans rendered to show higher density bone and dentition with false color (red) assigned to regions of higher density, including teeth along the jaw. *(d, f)* dorsal view, (*e*) lateral view. Abbreviations as in previous figures, rostral is to left in (*a-f*).

Figure 4. *Polyodon spathula*, *shh* expression in half-jaw mounts, oral surface, rostral at the top. (*a*) stage 42, *shh* is expressed as focal spots coincident with timing of initiation of tooth germs (arrow, see figure 2*p*). (*b*) stage 37, *shh* marking odontogenic field at on upper jaw and hyoid processes. (*c*, *d*) stage 39, *shh* loci marking 5 tooth germs on each quadrant. (*e*-*h*) stage 40, dermopalatine and dentary tooth fields are first to show *shh* loci. (*i*-*n*) *shh* loci increase in number and add first loci to1st hypobranchial (*k, lb*) in advance of any on the upper branchial arches (*n*). (*o, p*) stage 43, many more *shh* loci and at older stages (cap and cone stages), increase in number of tooth germs on lower jaw before upper jaw (*m*, *n*).

Figure 5. *Polyodon spathula* developmental series of *bmp4* expression in the dentition as half-jaw whole mounts ((*a*–*n*) stage 38 to 1dps)). (*a, a'*, *b*) lower jaw shows a band of mesenchymal expression associated with the overlying odontogenic band (arrowheads). (*c*-*n*) lower, upper jaw dentition showing *bmp4* expression in focal spots associated with condensed mesenchyme and papillary mesenchyme of each tooth position. (*h', i'*) new tooth positions appear caudally on the upper dermopalatine (arrowheads) and lower dentary, however, this tooth position addition changes in palatopterygoid (arrows) and hyoid tooth plates, where new tooth germs appear rostral to the functional row (as noted for *shh*, electronic supplementary information, figure 4), (*n, n'*) DAPI (white) counter-stained gelatin-albumin sections showing *bmp4* expression (digoxigenin, false colour, magenta) in mesenchymal papillary cells during tooth development on the hyoid (*n*, arrowheads) and upper and lower jaws (*n*'). Later in development during morphogenesis, weak expression of *bmp4* is present in the inner dental epithelium (*n'*, arrowheads).

Figure 6. *Polyodon spathula bmp4* gene expression in the developing dentition, stage 45. (*a*) Mid-sagittal section through the dentary (Meckel's cartilage) and hypobranchial 1. Expression is restricted to the dental mesenchyme and epithelium of developing teeth in the dentary (arrow; asterisk denotes taste bud in close proximity to Meckel's cartilage) and hypobranchial 1 (arrowhead). (*b*) Parasagittal section through the palatoquadrate and dermopalatine, with *bmp4* expression in both the inner dental epithelium (arrowhead) and papillary mesenchyme (arrow) of the cap-stage tooth, with the bone of attachment present (asterisk denotes taste bud). (*c*) Parasagittal section through the palatoquadrate, showing a functional (connected to the underlying

bone of attachment) first generation tooth and a neighbouring successional, but not replacing, tooth placode with *bmp4* expression in the dental epithelium (arrowhead; asterisk denotes taste bud). (*d*) Mid-sagittal section through hypobranchial 2, showing the functional tooth (dashed line) anterior to the epithelial expression of *bmp4* (arrowhead) associated with the developing adjacent successional tooth, not a replacement tooth; this shows the conservation of expression and similarity of proximity of adjacent teeth among multiple sites in the oropharynx of *P. spathula*. Abbreviations as in figure 1.

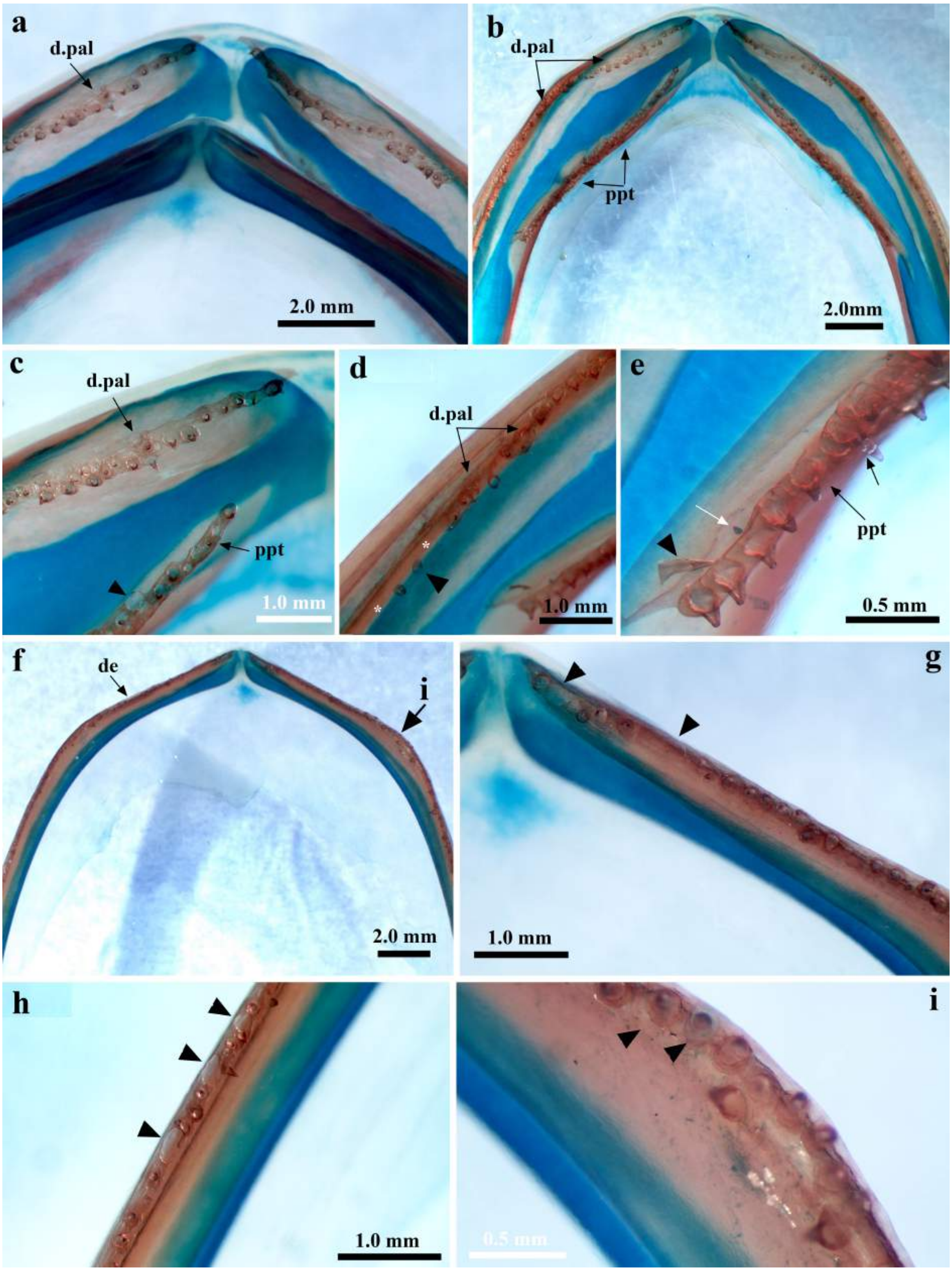
a
d.pal
2.0 mm
b
d.pal
ppt
2.0mm
c
d.pal
ppt
1.0 mm
d
d.pal
1.0 mm
e
ppt
0.5 mm
f
de
i
2.0 mm
g
1.0 mm
h
1.0 mm
i
0.5 mm

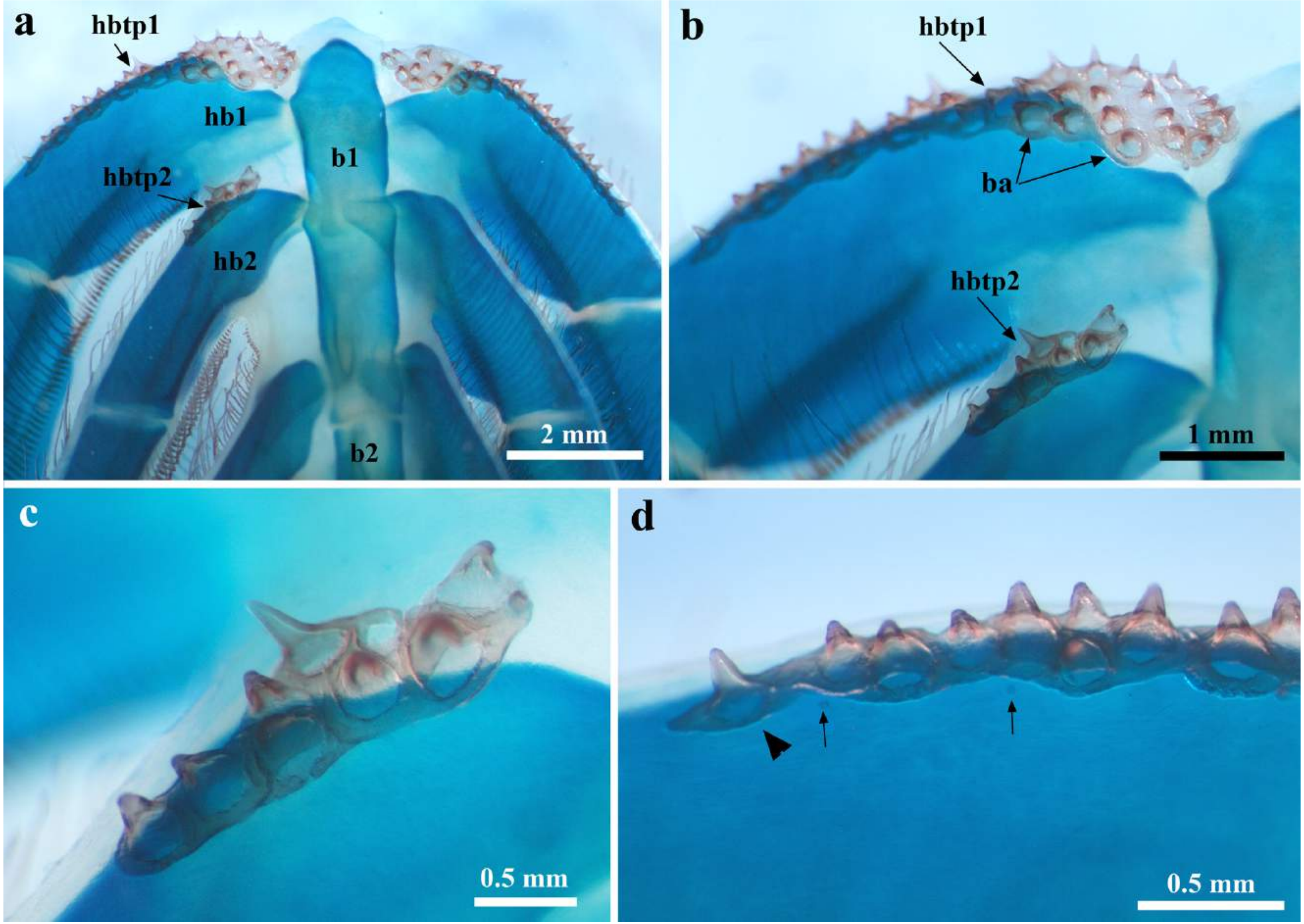
a
hbtp1
hb1
b1
hbtp2
hb2
b2
2 mm
b
hbtp1
ba
hbtp2
1 mm
c
0.5 mm
d
0.5 mm

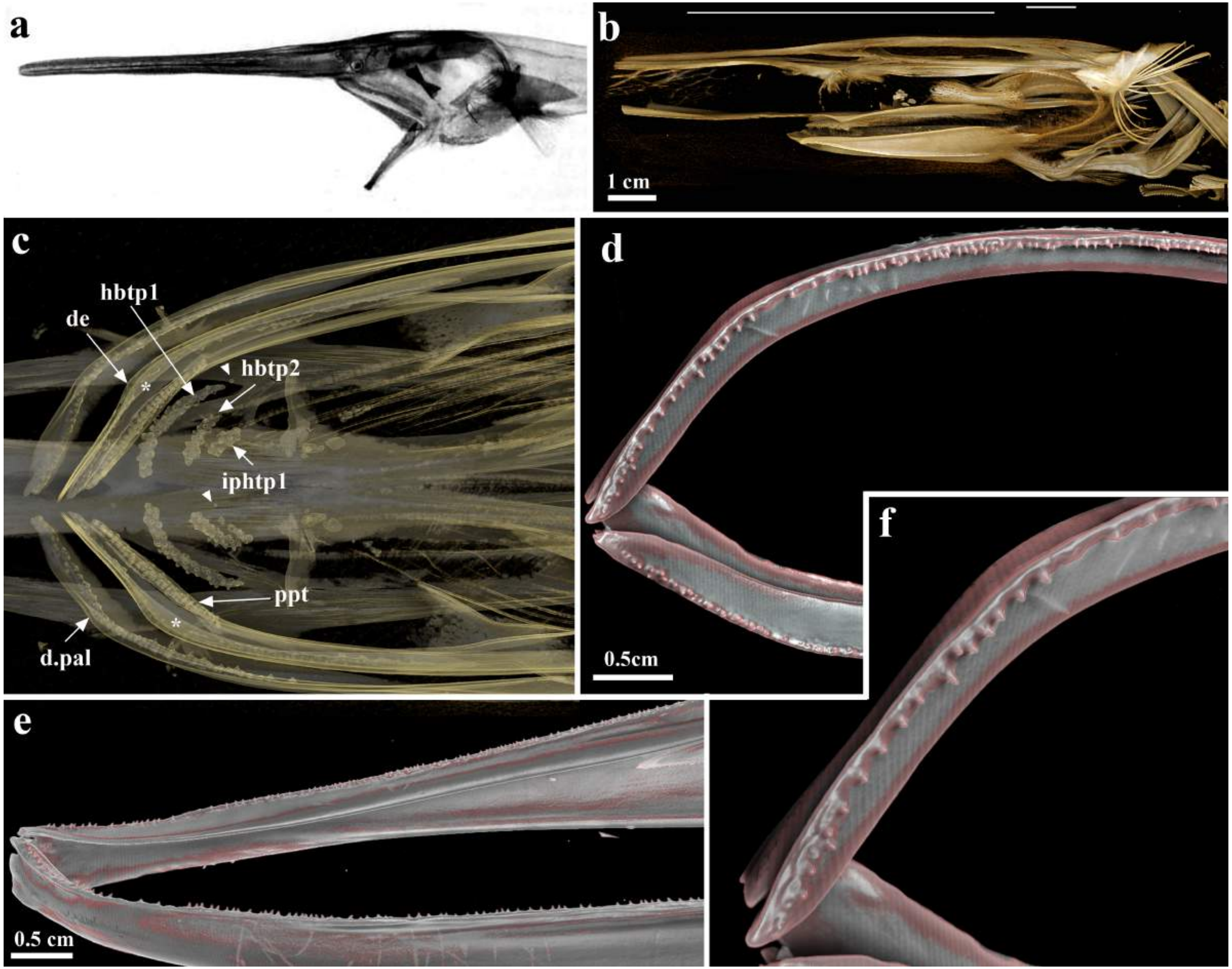

a
b
1 cm
c
hbtp1
de
hbtp2
iphtp1
ppt
d.pal
d
0.5cm
f
e
0.5 cm

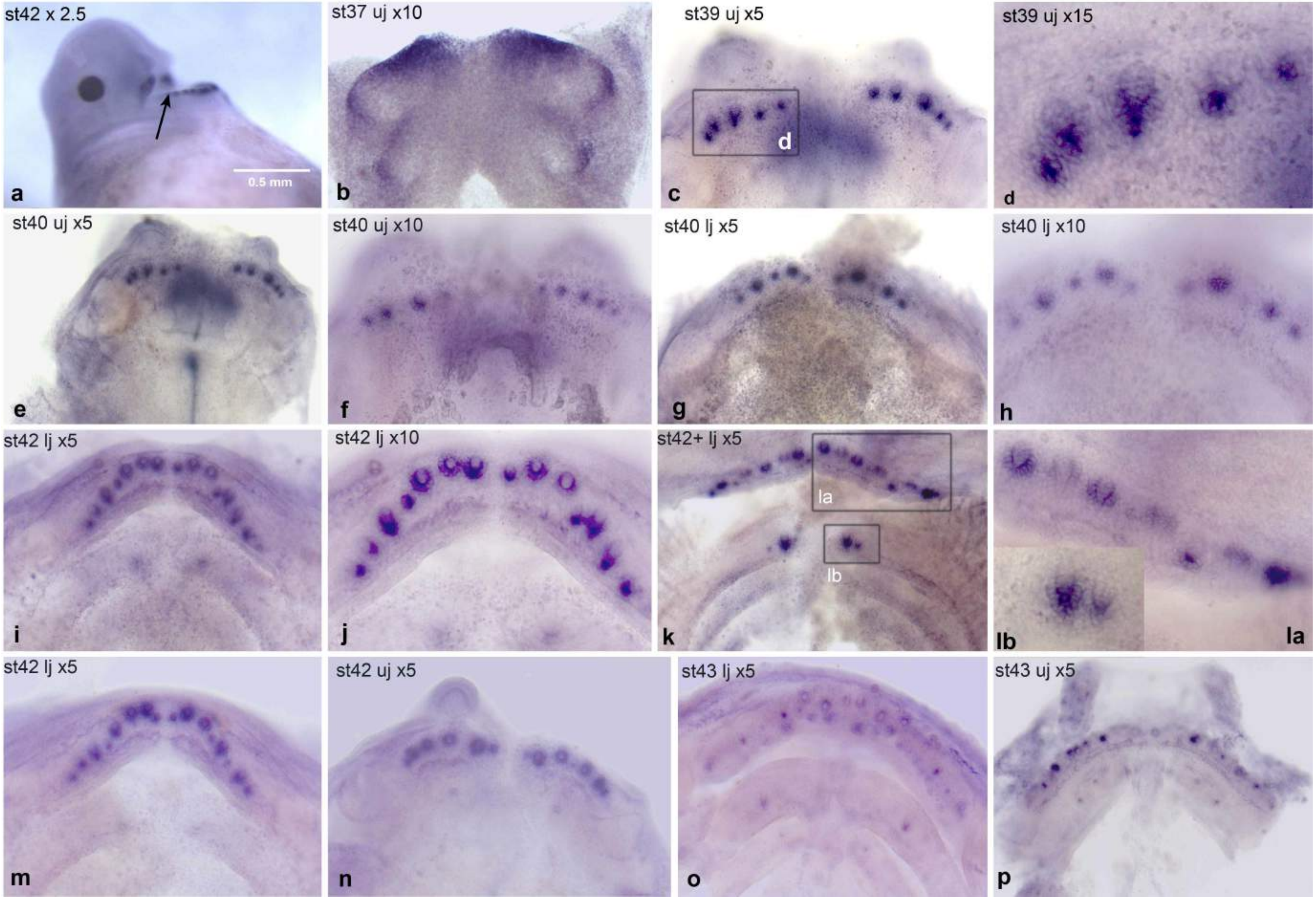

st42 x 2.5
0.5 mm
a
st37 uj x10
b
st39 uj x5
d
c
st39 uj x15
d
st40 uj x5
e
st40 uj x10
f
st40 lj x5
g
st40 lj x10
h
st42 lj x5
i
st42 lj x10
j
st42+ lj x5
la
lb
k
lb
la
st42 lj x5
m
st42 uj x5
n
st43 lj x5
o
st43 uj x5
p

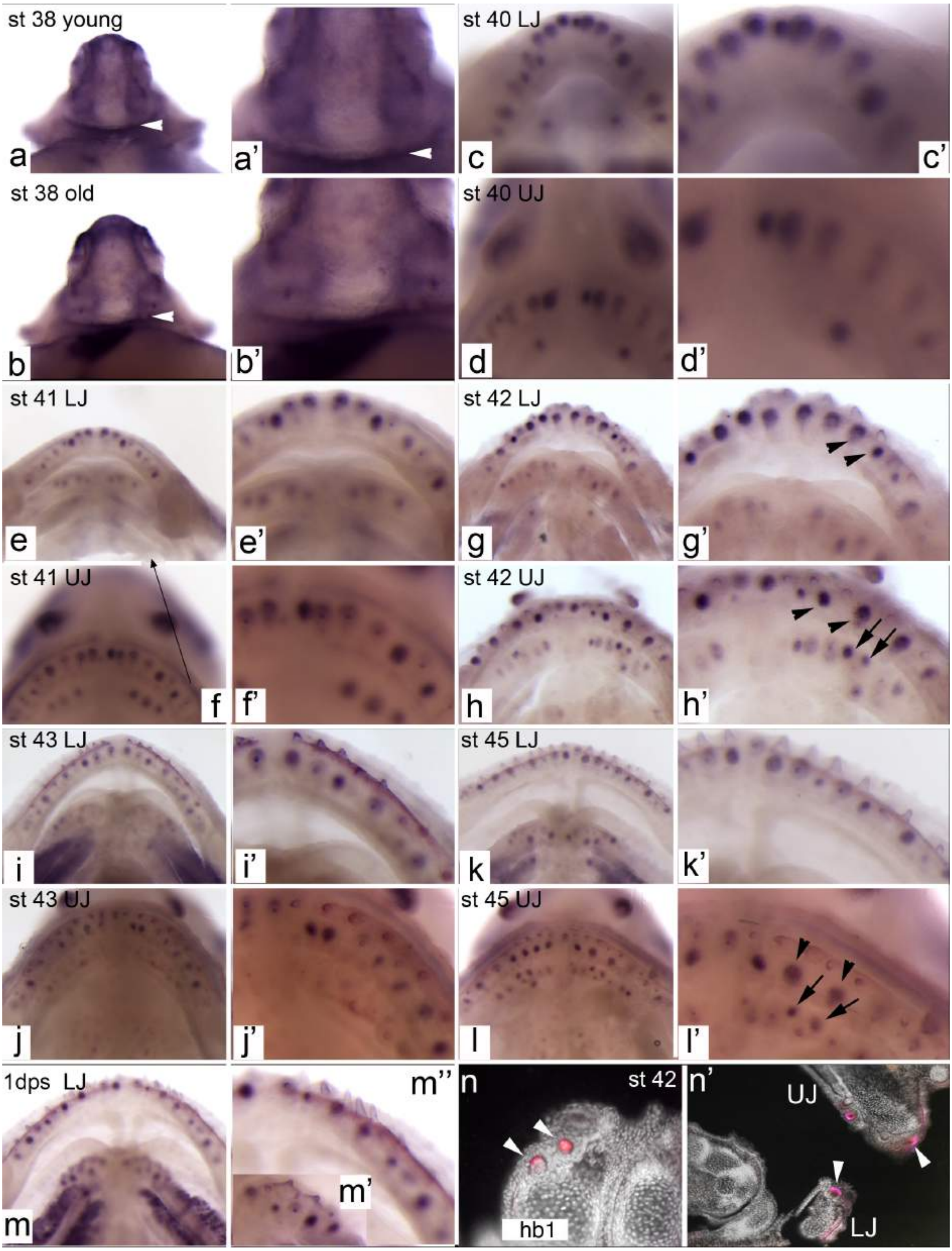

st 38 young
a
a'
st 40 LJ
c
c'
st 38 old
b
b'
st 40 UJ
d
d'
st 41 LJ
e
e'
st 42 LJ
g
g'
st 41 UJ
f
f'
st 42 UJ
h
h'
st 43 LJ
i
i'
st 45 LJ
k
k'
st 43 UJ
j
j'
st 45 UJ
l
l'
1dps LJ
m
m''
m'
n
st 42
hb1
n'
UJ
LJ

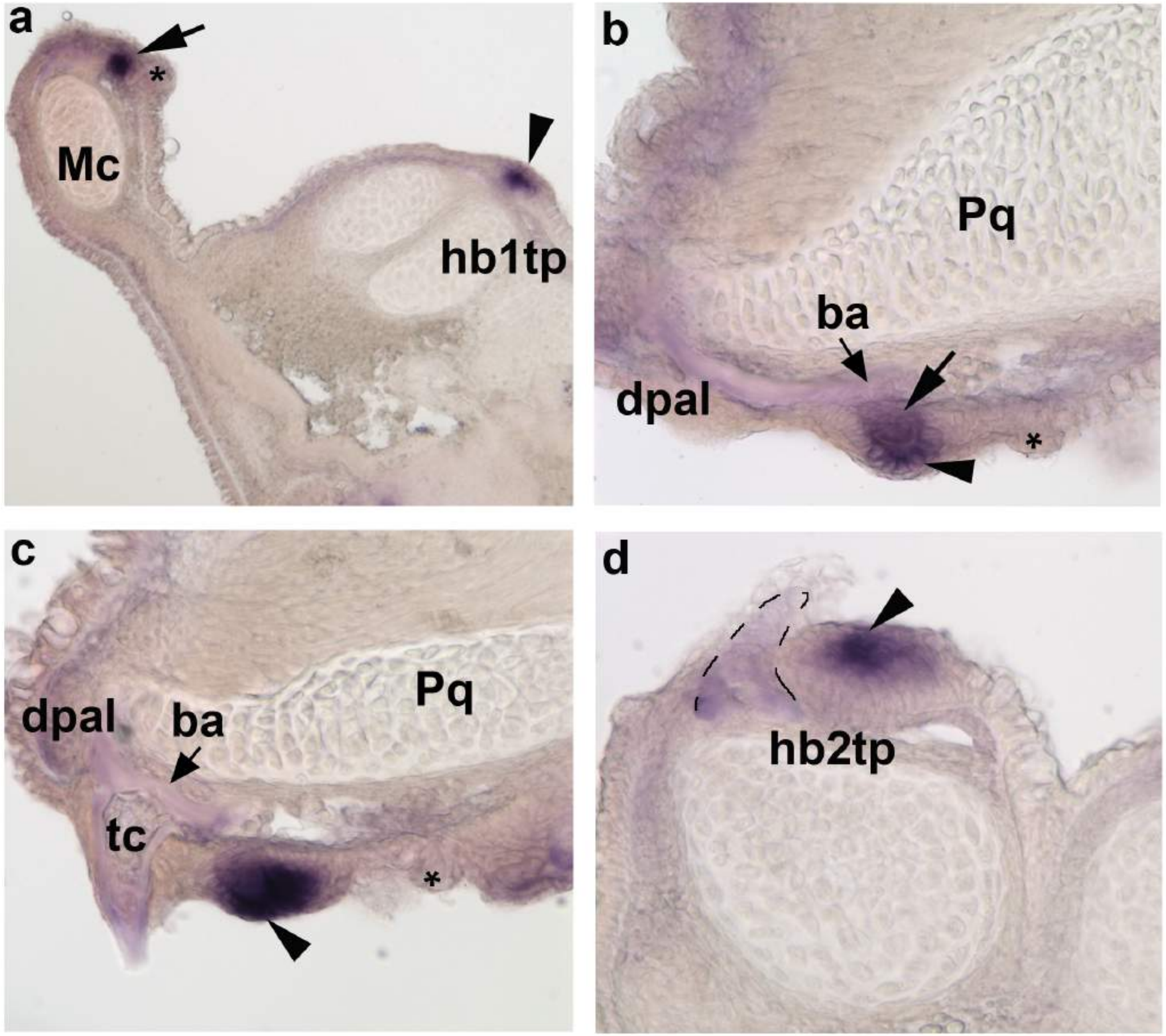
a
Mc
hb1tp
b
Pq
ba
dpal
c
dpal
ba
Pq
tc
d
hb2tp
